# Microbial single-cell omics: the crux of the matter

**DOI:** 10.1007/s00253-020-10844-0

**Published:** 2020-08-26

**Authors:** Anne-Kristin Kaster, Morgan S. Sobol

**Affiliations:** 1 Institute for Biological Interfaces 5, Karlsruhe Institute of Technology, Hermann-von-Helmholtz-Platz 1, D-76344 Eggenstein-Leopoldshafen, Germany; 2 Institute for Applied Biosciences, Karlsruhe Institute of Technology, Fritz-Haber-Weg 4, D-76131 Karlsruhe, Germany

**Keywords:** Targeted cell sorting, Amplification, Bioinformatics, Bacteria, Archaea

## Abstract

**Abstract:**

Single-cell genomics and transcriptomics can provide reliable context for assembled genome fragments and gene expression activity on the level of individual prokaryotic genomes. These methods are rapidly emerging as an essential complement to cultivation-based, metagenomics, metatranscriptomics, and microbial community-focused research approaches by allowing direct access to information from individual microorganisms, even from deep-branching phylogenetic groups that currently lack cultured representatives. Their integration and binning with environmental ‘omics data already provides unprecedented insights into microbial diversity and metabolic potential, enabling us to provide information on individual organisms and the structure and dynamics of natural microbial populations in complex environments. This review highlights the pitfalls and recent advances in the field of single-cell omics and its importance in microbiological and biotechnological studies.

**Key points:**

*• Single-cell omics expands the tree of life through the discovery of novel organisms, genes, and metabolic pathways.*

*• Disadvantages of metagenome-assembled genomes are overcome by single-cell omics.*

*• Functional analysis of single cells explores the heterogeneity of gene expression.*

*• Technical challenges still limit this field, thus prompting new method developments.*

## Introduction

### Microbial dark matter (MDM)

Prokaryotic microorganisms harbor an enormous potential for biotechnological applications, such as novel natural product discovery, bioenergy production, and bioremediation of harmful anthropogenic-introduced substances (Singh et al. 2014; Katz and Baltz 2016; Kumar and Kumar 2017; Mullis et al. 2019; Stincone and Brandelli 2020). However, despite their global quantity and importance, it is estimated that over 99% of all microbial species remain uncultured or even completely uncharacterized (Wu et al. 2009; McDonald et al. 2012; Hug et al. 2016; Lloyd et al. 2018). These microorganisms are referred to as microbial dark matter (MDM). Very often, attempts to grow microbes under laboratory conditions fail, or they grow too slowly to obtain sufficient biomass for analysis. Although new cultivation methods have been recently developed (Nichols et al. 2010; Sherpa et al. 2015; Wiegand et al. 2020), genome sequences for the vast majority of prokaryotes have been inaccessible, obscuring our knowledge of microbial diversity, metabolism, (eco)physiology, inter-organism interactions, and adaptive evolution.

### Cultivation-independent approaches for exploring MDM

The development of shotgun sequencing of DNA extracted from environmental samples—so-called metagenomics—has provided extensive gene content information from natural microbial communities (Temperton and Giovannoni 2012; Hedlund et al. 2014; Anantharaman et al. 2016; Vollmers et al. 2017a; Quince et al. 2017). Unfortunately, assembling individual discrete genomes from metagenomics data (so-called MAGs, metagenome-assembled genomes) is often difficult and rather costly, especially for high diversity samples or low abundant organisms (Gilbert and Dupont 2011; Narasingarao et al. 2012; Iverson et al. 2012). Binning algorithms, which group contigs and assign them to operational taxonomic units (OTUs), have massively improved over the past years, but MAGs are still likely consensus genomes derived from multiple cells that are equally abundant and share high-homologous regions (Venter et al. 2004; Rusch et al. 2010; Iverson et al. 2012). Large structural variants (SVs) such as genome rearrangements, gene insertions, duplications, or losses which can vary in highly homologous cells due to mutations, horizontal gene transfer (HGT), and recombination cannot be analyzed and mobile genomic elements such as plasmids or transposable elements cannot be accurately binned (Fraser et al. 2007; Vergin et al. 2007; Martinez-Garcia et al. 2012b; Shapiro et al. 2012; Dam et al. 2020). In recent years, long-read sequencing technologies such as Pacific Biosciences’ (PacBio) single-molecule real-time (SMRT) sequencing and Oxford Nanopore Technologies’ (ONT) nanopore sequencing have improved the detection and study of large SVs that lead to heterozygosity on the strain level (Amarasinghe et al. 2020; Ho et al. 2020). However, these technologies are sometimes still limited in use due to the lack of efficient extraction of high-molecular-weight nucleic acids and required library input amounts, as well as higher error rates (Frank et al. 2016; Amarasinghe et al. 2020).

Analyzing the RNA of a microbial community—so-called metatranscriptomics—can provide information of the actual gene expression, function, and metabolic activity given enough sequencing depth and repetition of sequenced samples. It even allows the determination of relative expression levels between active genes, making it a useful complement to metagenomics for studying ecological and metabolic interactions within microbial communities (Shi et al. 2011; Mason et al. 2012). However, even isogenic microbial populations show substantial cell-to-cell heterogeneities in transcriptional activity (Roberfroid et al. 2016; González-Cabaleiro et al. 2017). Since genomic heterogeneity is a common characteristic of microorganisms to adapt to environments with constant and rapid changes, standard metagenomic and metatranscriptomic analyses alone are not always well suited to obtain genomes of different species and deliver unequivocal information about the organization and activity of discovered genes within genomes, making it difficult to predict functional genes, metabolic pathways, and potential benefits of microbial species. This holds especially true for microorganisms with low abundance in a habitat since the quality of genome reconstruction is largely dependent on sequence coverage for assembly as well as coverage covariance-based binning (Albertsen et al. 2013; Vollmers et al. 2017b; Dam et al. 2020).

## Omics on the single-cell level

**Fig. 1:**
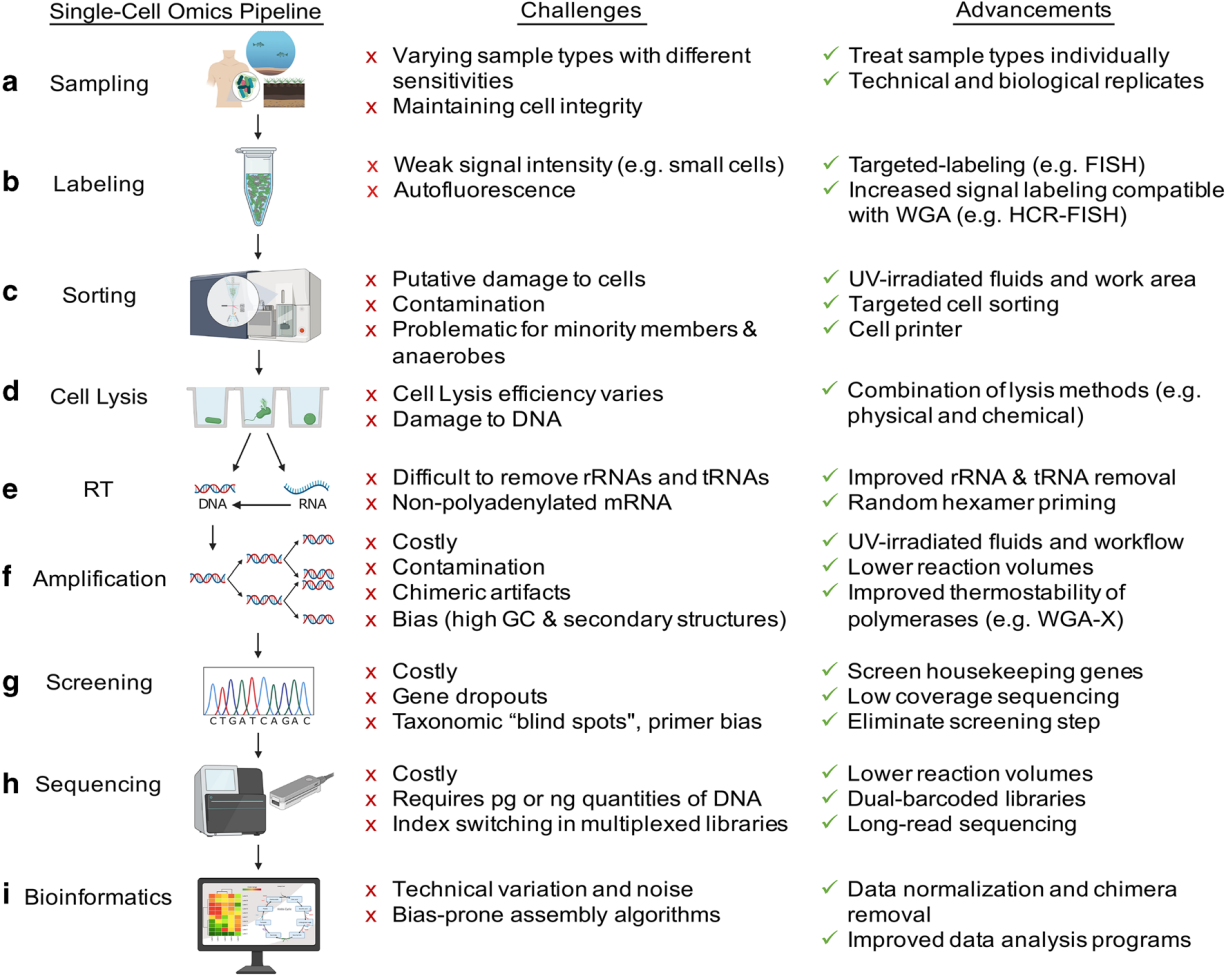
Overview of a single-cell omics pipeline including respective challenges and advancements. **a** Unless analyzed immediately, environmental samples require deep-freezing in the presence of a cryoprotectant that preserves the integrity of the cell and its nucleic acids. **b** Cells are stained with a fluorescent dye, such as DAPI or SYBR® Green; however, they can also be specifically labeled. **c** Physical isolation of a single cell is typically performed by fluorescence-activated cell sorting (FACS) into multi-well plates. **d** After separation, the single cells are lysed to release their DNA and RNA. Today, most cell lysis in SC omics relies on an alkaline solution. **e** For single-cell transcriptomics (SCT), RNA must first be converted to double-stranded cDNA via reverse transcription prior to amplification. **f** Since a typical prokaryotic cell only contains a few fg grams of DNA and RNA, whole genome or transcriptome amplification (WGA/WTA) is needed by a factor of about 10^6^. Multiple displacement amplification (MDA) is the most widely used reaction. **g** PCR is used to screen for specific loci after amplification, usually with broad eubacterial and archaeal 16S rRNA primers, followed by Sanger sequencing. **h** After DNA or RNA library preparation, next-generation sequencing technologies like Illumina, Oxford Nanopore, or PacBio are available for genome/transcriptome sequencing. **i** After quality assessment, trimming, and/or normalization of the sequencing reads, bioinformatics tools can conduct the assembly, ORF calling, and annotation of the genes, as well as pathway reconstruction and gene comparisons. Created with BioRender

**Fig. 2:**
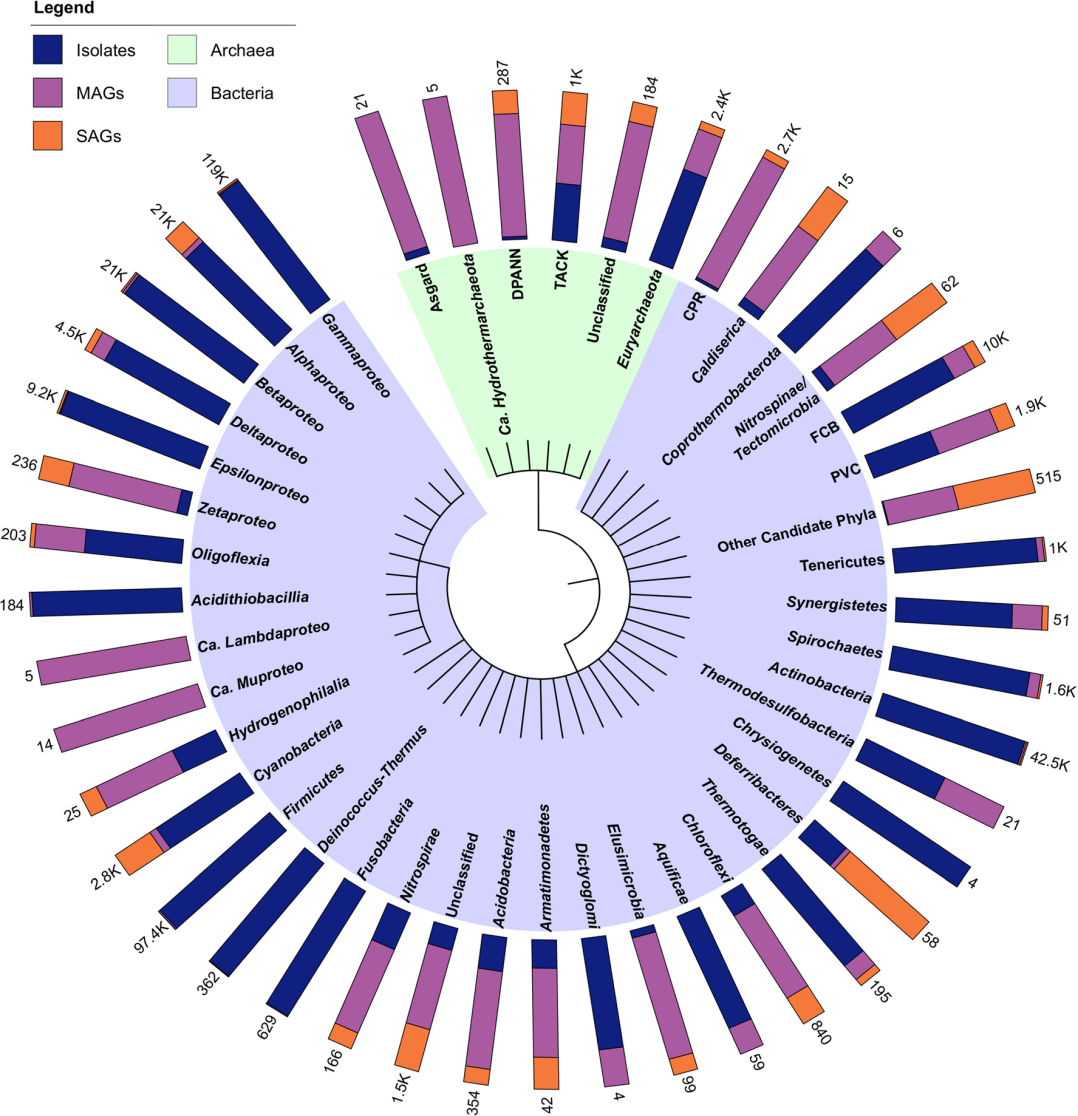
Cladogram of prokaryotes (Bacteria and Archaea) showing the relative proportions of isolate genomes, single-amplified genomes (SAGs), and metagenome-assembled genomes (MAGs) that make up the total number of genomes in each phylum. The taxonomy is based on National Center for Biotechnology Information (NCBI) (Sayers et al. 2020). Total genome numbers for each phylum are shown at the top of each bar. Data extracted from the Genomes OnLine Database (GOLD) in July 2020 (Mukherjee et al. 2019). Cladogram created with Interactive Tree of Life (iTOL) version 5 (Letunic and Bork 2019). *-proteo* -*proteobacteria*. *Asgard Lokiarchaeota*-*Thorarchaeota*-*Odinarchaeota*-*Heimdallarchaeota*. *DPANN Diapherotrites*-*Parvarchaeota*-*Aenigmarchaeota*-*Nanoarchaeota*-*Nanohaloarchaeota*. *TACK Thaumarchaeota*-*Aigarchaeota*-*Crenarchaeota*-*Korarchaeota*. *FCB Fibrobacteres*-*Chlorobi*-*Bacteroidetes*. *PVC Planctomycetes*-*Verrucomicrobia*-*Chlamydiae*. *CPR Candidate Phyla Radiation*

Single-cell genomics (SCG) has emerged as a powerful technique to overcome disadvantages of genome reconstruction from metagenomes, by enabling genome analysis of an individual prokaryotic cell, referred to as single amplified genomes (SAGs) (Woyke et al. 2010; Stepanauskas 2012; Blainey 2013; Kaster et al. 2014; Woyke et al. 2017; Sewell et al. 2017; Piel and Cahn 2019). It allows for the physical separation of single cells directly from environmental samples, followed by sequencing and assembly of their individual genomes and consists of a series of integrated processes (Fig. 1). Since its first application on microorganisms in 2005 (Raghunathan et al. 2005), SCG has become a powerful tool for studying uncultivable organisms and delineating complex populations. An increasing number of SAGs are available from public databases such as the National Center for Biotechnology Information (NCBI) GenBank (Sayers et al. 2020), and/or the Joint Genome Institute Genomes OnLine Database (GOLD) (Mukherjee et al. 2019), which includes all data from Integrated Microbial Genomes (IMG). As of July 2020, over 9000 SAG sequencing projects have been deposited in GOLD (Mukherjee et al. 2019), of which many are classified as uncultured and potentially novel taxonomic groups (Swan et al. 2011; McLean et al. 2013; Hedlund et al. 2014; Becraft et al. 2016; León-Zayas et al. 2017; Landry et al. 2017) (Fig. 2). Recently, a new reference database containing over 12,000 SAGs from the euphotic ocean was published (Pachiadaki et al. 2019), greatly expanding our knowledge on the diversity and complexity of marine microorganisms. SAGs of some species are however still difficult to obtain, for example, minority members in a microbial community or anaerobic organisms. This could be due to the difficulty in labeling them (Müller and Nebe-Von-Caron 2010) and/or the increased chance that they will lyse when exposed to oxygen, risking the DNA to be damaged during lysis steps prior to the downstream applications of SCG. In addition, the success of genome recovery from single cells, in general, varies widely from 0% to a finished genome.

## Technical problems, recent advances, and future challenges

In general, the workflow involves (a) sampling and preservation, (b) non-specific staining of microbial populations, (c) cell sorting, (d) cell lysis, (e, f) whole genome amplification (WGA), (g) screening for SAGs of interest, and (h, i) sequencing and analysis (Fig. 1). For cell isolation, several methods can be used such as microfluidics, micromanipulation, and—conventionally—fluorescence-activated cell sorting (FACS). Microfluidic- and optofluidic-based systems (Marcy et al. 2007b; Marcy et al. 2007a; Blainey et al. 2011; Landry et al. 2013; Gole et al. 2013; Xu et al. 2016; Riba et al. 2016; Lan et al. 2017), as well as micromanipulation (Woyke et al. 2010; Grindberg et al. 2011), have the advantage of sorting cells based on their morphology and less physical stress applied to the cell. Most setups even allow for cell separation, lysis, and amplification performed in one closed system at nanoliter or even picoliter volumes (Marcy et al. 2007b; Marcy et al. 2007a; Blainey et al. 2011; Landry et al. 2013; Xu et al. 2016); however, some limitations remain in cell throughput and the successful recovery of non-contaminated amplified products. FACS has become the most commonly used method for separation due to its high speed and flexibility with the use of different fluorescence signals (Stepanauskas and Sieracki 2007; Rinke et al. 2014; Woyke et al. 2017). The main limitations of this technology include potential contamination due to the open-plate workflow, the inability to microscopically examine cells, the strong physical stress applied to cells, and further miniaturization of downstream reaction volumes (Blainey 2013; Woyke et al. 2017).

Cell lysis efficiency plays an important role in the success rate of SCG owing to the challenging natural diversity of microbial cell walls. Environmental sample preservation has a significant impact on lysis, and conditions have to be optimized for each organism of interest (Stepanauskas 2012; Rinke et al. 2014). Cell lysis methods need to accomplish releasing DNA and RNA from the cell while maintaining its integrity (Clingenpeel et al. 2014a) and must not interfere with downstream reactions. Alkaline lysis is the most widely used method; however, more efficient lysis of cells from complex communities can be accomplished by using a combination of different lysis methods (i.e., physical-like freeze-thaw cycles, chemical, or enzymatic) (Hall et al. 2013; He et al. 2016; Stepanauskas et al. 2017).

In general, WGA via multiple displacement amplification (MDA) to obtain a sufficient quantity of genomic DNA for sequencing remains the major limitation of the SCG pipeline. In addition to the high costs, this method often results in incomplete and uneven genome amplification and is biased against high GC regions of the genome (Lasken and Stockwell 2007; Lasken 2009; Sabina and Leamon 2015). Therefore, the average completeness of genomes obtained by SCG is often lower than that of MAGs from the same sample (Blainey et al. 2011; Bowers et al. 2017; Landry et al. 2017; Alneberg et al. 2018; Dam et al. 2020). One of the main sequencing limitations is due to MDA artifacts such as chimeras, uncontrolled bias, and non-specific products (Lasken and Stockwell 2007; Sabina and Leamon 2015). Amplification bias is thought to occur predominantly by random mechanisms, as sequences that are over-represented in one MDA reaction can be under-represented in another. However, there are some reproducible differences in the average level of representation of different sequences, and certain sequences are not amplified at all for unknown reasons (Dean et al. 2001; Lasken and Stockwell 2007; Lasken 2009). Several approaches can be taken to lessen the amplification bias of the MDA reaction such as reducing the reaction volume to improve the specificity of the reaction (Marcy et al. 2007a; Landry et al. 2013; Leung et al. 2016; Yu et al. 2017; Lan et al. 2017), post-amplification endonuclease treatment to reduce chimeric sequences (Zhang et al. 2006), and post-amplification normalization by nuclease degradation of dsDNA to reduce most abundant sequences (Rodrigue et al. 2009). To overcome the problem of amplification bias against high GC content, a more thermotolerant phi29 DNA polymerase was recently described, resulting in higher quality draft genomes of single sorted cells (Stepanauskas et al. 2017). The hybrid amplification method called multiple annealing and looping-based amplification cycles (MALBAC) can also amplify high GC regions and showed amplification of more than 90% of the genome of single human cells; however, the method is very costly and not that suitable for high-throughput applications for prokaryotes. Many amplification methods have been compared in the past, but the general consensus is that the amplification method of choice is dependent on the questions being asked as the methods each have their distinct advantages and disadvantages (De Bourcy et al. 2014; Huang et al. 2015; Estévez-Gómez et al. 2018).

### Low throughput for low abundant cells: phylogeny-driven targeted labeling

Due to the amplification bias, statistically less than 30% of the 16*S* rRNA gene gets amplified (Swan et al. 2011). This makes screening challenging, especially for rare organisms of the population or cells that are hard to lyse. Like metagenomics, SCG is therefore often not a cost-efficient approach to unravel microbial community members which are not abundant, especially in complex environments. Given the limitations of cultivation-dependent and cultivation-independent techniques, minority members of microbial communities are therefore often overlooked and understudied. Nevertheless, they may still play important roles in many biogeochemical processes (e.g., due to high enzyme affinities to certain substrates) or might have biotechnological relevance (Frias-Lopez et al. 2008; Shi et al. 2011; Pratscher et al. 2018). Generating samples enriched for the specific microorganism of interest will increase the likelihood of actually sorting those cells and obtaining positive amplifications, which will ultimately reduce experimental costs.

Recently, Dam et al. (2020) implemented a fluorescent in situ hybridization (FISH) labeling, coupled with targeted cell sorting to obtain genomes of low abundant community members of the phylum *Chloroflexi* using a modified protocol (Podar et al. 2007; Yilmaz et al. 2010; Haroon et al. 2013). Usually, fluorescent labeling of bacterial cells requires fixatives such as paraformaldehyde to increase the fluorescent signal by allowing stronger permeabilization of the cell membrane and penetration of the probe. However, since the process weakens the cell wall, it can lead to the lysis of the labeled cells during the sorting process. Furthermore, paraformaldehyde compromises the downstream applications for SCG, namely, the amplification of the genomic DNA via MDA (Clingenpeel et al. 2014b; Doud and Woyke 2017). Dam et al. (2020) demonstrated that an in-solution, fixation-free FISH protocol allowed phylogenetic labeling of targeted cells to remain intact during the sorting process and that their fluorescent signals were sufficiently high for multiple sorts. This was achieved by longer hybridization times and higher probe concentrations to overcome the problem of low cell membrane permeability when no additional fixatives and lysing agents could be used.

Furthermore, catalyzed reporter deposition (CARD)-FISH (Pernthaler et al. 2002) could overcome weak FISH signals, which occur if the cells are in a low activity state, as is the case for most samples from oligotrophic environments. In some cases, the enrichment of low abundant bacterial cells after CARD-FISH and FACS doubled in comparison to the original sample (Wallner et al. 1997; Sekar et al. 2004). Through the use of CARD-FISH and laser microdissection, researchers were able to confirm the previously unknown identity of a bacterium that produces the cytotoxin calyculin, by using the biosynthetic gene cluster as the probe (Wakimoto et al. 2014). However, like with traditional FISH, the paraformaldehyde used is not compatible with whole genome amplification, which is why multiple cells were needed to avoid the amplification step. To combat this issue but still maintain comparable signal intensities, a modified hybridization chain reaction (HCR)-FISH protocol which does not require paraformaldehyde as a fixative was recently used and combined with FACS and MDA to successfully enrich bacteria from an environmental sample (Yamaguchi et al. 2015b; Yamaguchi et al. 2015a; Grieb et al. 2020).

Another labeling tool for gene-specific targeting is recognition of individual genes (RING)-FISH which can be combined with targeted cell sorting (Pratscher et al. 2009). This technique was used to target a previously unculturable methane oxidizer in soils—USCα—(Kolb et al. 2005), where the specific methane monooxygenase enzyme (pMMO) was known, but the 16S rRNA gene sequence was not. Application of a fluorescently labeled suicide substrate for pMMO was used to specifically sort the labeled cells via FACS and obtain the 16S rRNA sequence. For the first time, 16S rRNA FISH allowed then a direct link to the high-affinity activity of methane oxidation by USCα cells in situ. Analysis of the global biogeography of this group by 16S rRNA then further revealed its presence in previously unrecognized habitats, such as subterranean and volcanic biofilm environments, indicating a potential role of these environments in the biological sink for atmospheric methane.

### Cherry-picking: function-driven single-cell genomics

Recently, different techniques for functional-driven SCG have been used to select and characterize single cells based on a specific functional trait prior to and in conjunction with WGS (Woyke and Jarett 2015; Lee et al. 2015; Doud and Woyke 2017; Couradeau et al. 2019; Hatzenpichler et al. 2020). Some of these methods have provided important ecological and biotechnological findings, which help to expand our knowledge on previously unknown gene functions (Woyke et al. 2018).

Raman micro-spectroscopy in combination with cell sorting, called Raman activated cell ejection (RACE), has been used to provide phenotypical and biochemical information of single cells without damaging the cells (Huang et al. 2004; Huang et al. 2010; Li et al. 2012). Song et al. (2017b) reported the first use of RACE coupled with SCG to detect novel carotenoid and isoprenoid synthesizing bacteria from the Red Sea as a label-free approach (Song et al. 2017b). They later showed that stable isotope labeling with D_2_O in combination with RACE and WGA could detect antimicrobial-resistant bacteria in the Thames River (Song et al. 2017a).

Substrate analog probing (SAP) uses synthetic molecules that are similar to naturally occurring molecules to study substrate uptake in organisms. This method has been combined with FACS and SCG by the use of fluorescently labeled substrates to uncover the significance of polysaccharide degradation by *Verrucomicrobia* (Martinez-Garcia et al. 2012a) and a novel cellulose-degrading bacterium from the candidate phylum *Goldbacteria* with less than 1% abundance in the sample (Doud et al. 2019). However, the greater use of this method is mainly limited by the uncertainty in how/if the fluorescent tag interferes with enzyme-substrate binding (Hatzenpichler et al. 2020). Bio-orthogonal non-canonical amino acid tagging (BONCAT) is one approach that has been used to overcome this limitation because detecting the uptake of these molecules is independent of the labeling approach. In general, this method uses synthetic amino acids that are taken up by bacterial cells so that they are incorporated into newly synthesized proteins which then become fluorescent due to azide-alkyne click chemistry (Mahdavi et al. 2014; Hatzenpichler et al. 2014). BONCAT has been previously used to identify pathogenic bacteria and their secreted proteins (Mahdavi et al. 2014; Sherratt et al. 2017). Recently, this targeting method was also shown to be compatible with FACS and MDA which enabled the authors to sort methane-oxidizing archaea-bacterial consortia from deep sea sediments and identify in situ activity and novel interactions between these organisms (Hatzenpichler et al. 2016). However, the current limitations, such as signal strength, substrate analog specificity, and quantification of activity rates, restrict the widespread use of these methods and need to be further improved for high-throughput applications (Doud and Woyke 2017; Hatzenpichler et al. 2020).

### Viral-host interactions in single-cells

Studying viral-host interactions via metagenomics has proven to be difficult due to the naturally high diversity in most environments. Studies comparing MAGs and SAGs from the same samples find that the MAGs do not contain a significant portion of genes, indicative of prophage infection found within the SAGs, even within largely incomplete SAGSs (Labonté et al. 2015; Dam et al. 2020). SCG has therefore revolutionized the study of viruses in many environments, as well as their interactions within individual prokaryotic cells. In the marine subsurface, where cell abundance and cell activity is low, a particular Firmicute, *Desulforudis*, interestingly undergoes frequent HGT and viral infections (Labonté et al. 2015). Also, up to 32% of genes that were recovered from SAGs, were not present in the metagenome of the same sample. Munson-Mcgee et al. (2018) studied viral-host interactions within a hot spring environment and found that 60% of SAGs contained at least one virus, showcasing the broad host range of viruses (Munson-Mcgee et al. 2018). The authors also estimated that for low-level completed SAGs, only a 300 bp viral sequence minimum is needed to detect a virus-host interaction. By directly sequencing single viral genomes (vSAGs) from the Mediterranean Sea and the Atlantic Ocean, highly abundant and ecologically significant viruses could be discovered, which had been previously missed through metagenomics (Martinez-Hernandez et al. 2017). When the vSAGs were compared to public metagenome datasets, they found that their vSAGs best represent the diversity of viruses in global oceans. Notably, a large portion of viral diversity has still gone undiscovered in the human virome. De La Cruz Peña et al. (2018) found that most publicly available viral isolates did not match vSAGs in the oral virome. The authors also found that the viral community was highly variable and interpersonal between patients because none of the vSAGs obtained in this study were found in all viromes.

### The next step: single-cell transcriptomics (SCT)

Although SCG has been successfully applied in microbial ecology studies, it only provides information related to genetic architecture and metabolic potential of a cell, but does not reveal the actual gene expression and activity of a cell under different environmental conditions. This, and the fact that the transcriptomic profile of individual cells can vary even if they are genetically homogenous underlines the necessity of studying the transcriptomic profile at single-cell resolution (de Jager and Siezen 2011; Kanter and Kalisky 2015). However, compared to genomic analysis, transcriptomic analysis for microorganisms at the single-cell level is much more challenging. Single-cell transcriptomics (SCT) in eukaryotic organisms has already been successfully applied (Kanter and Kalisky 2015; Tsang et al. 2015; Fan et al. 2015). Cells are first individually sorted and lysed; then, the RNA is converted to cDNA with oligo (dT) primers and amplified prior to library preparation and sequencing. Whilst a single eukaryotic cell contains up to 50 picograms (pg) of total RNA (Kang et al. 2011), the RNA concentration in prokaryotes is typically in the low femtogram range (1–5 fg) (Kang et al. 2011; Wang et al. 2015). Unfortunately, only a small fraction of total RNA is mRNA, as rRNA and tRNA molecules usually represent over 90% of the total RNA. Since most SCT methods target polyadenylated transcripts, the lack of a polyA tail on most mRNAs in prokaryotes means that the large fraction of rRNA and tRNA cannot be depleted using the methods established for eukaryotic transcriptomics. Furthermore, this structural difference of the mRNA molecule and the fact that prokaryotic mRNA can be polycistronic explains why not all methods for eukaryotic SCT can be applied to microorganisms. In addition, mRNA molecules have low stability over time. Nevertheless, in the recent past, several new approaches have been developed for SCT studies in prokaryotic cells (Kang et al. 2011; Kang et al. 2015; Wang et al. 2015; Liu et al. 2019). One method developed a rolling circle amplification (RCA) protocol that used the phi29 polymerase and random primers to amplify transcripts from single *Burkholderia thailandensis* cells (Kang et al. 2011). Even though this method was only used with microarray analysis, the authors report that including an rRNA depletion step would make this protocol compatible with next-generation sequencing (Kang et al. 2011; Kang et al. 2015). Another method combined SCT with RNA-seq, where RNA-based, single primer isothermal amplification (Ribo-SPIA) was used to amplify transcripts from *Synechocystis* sp. PCC 6803 (Wang et al. 2015). The authors used this method to show cell heterogeneity under environmental stress due to nitrogen starvation and successfully detected up to 98% of all putative genes within the genome. The most recently published method used RNA-seq in combination with whole transcriptome amplification (WTA) with the REPLI-g WTA kit (Qiagen) to amplify total RNA from *Porphyromonas somerae* (Liu et al. 2019). However, only 0.3% of rRNA transcripts were detected, which might have been due to the slow growth rate of *P*. *somerae*.

In general, these studies already provide the basic framework for SCT; however, they have so far only been carried out on model organisms, and many technical challenges still remain such as the inefficient transcriptome coverage, biased amplification, and throughput (Picelli 2017; Chen et al. 2017; Zhang et al. 2018). Direct-RNA methods that do not require pre-amplification, e.g., Oxford Nanopore sequencing systems, would be ideal to cut out amplification bias (Garalde et al. 2018). This method however still requires at least 100 nanograms (ng) of RNA and is based on polyadenylated mRNA transcripts. Nevertheless, it is possible that future improvements will decrease the input amount of RNA needed, making the technique more applicable for single-cell analysis.

### Back to multiple cells?

Until improved technologies become available, completing the assembly of the microbial genomes and transcriptomes for most species likely requires a combination of approaches such as reconstruction from multiple homologous cells if available or additional metagenomic data. Metagenomic reads and contigs can be used to recover missing genomic regions and greatly improve the SAG assemblies (Dodsworth et al. 2013; Nurk et al. 2013; Becraft et al. 2016; Xu and Zhao 2018). Likewise, metagenomic bins can also be improved by SAG datasets (Pachiadaki et al. 2019). Another approach is the so-called mini-metagenomic approach, where a few cells (50–1000) are sorted into one well (Yu et al. 2017; Schulz et al. 2018; Alteio et al. 2020; Geesink et al. 2020; Grieb et al. 2020). Sorting multiple cells of the same type by targeted labeling might circumvent the necessity of additional amplification of DNA/RNA, therefore allowing single genomes/transcriptomes to be bioinformatically binned from those mini-metagenomes. This not only reduces the cost but also lessens the chance for contamination and increases genome coverage (Podar et al. 2007; Yu et al. 2017; Grieb et al. 2020) and might be a sufficient solution for some biological questions.

## Outlook

The integration and binning of SCG with environmental omics data can already provide unprecedented insights into microbial diversity, metabolic features, and processes. SCG and SCT have tremendous potential to bring more clarity to the contested discussion about the nature of prokaryotic species and their metabolic potentials which will enable us to provide information on individual organisms and the structure and dynamics of natural microbial populations in all kinds of environments. SC omics holds great promise in microbial microevolution studies, industrial bioprospecting, and selection of suitable heterologous expression systems, with potential for novel and environmentally responsible energy solutions, bioremediation of toxins, and natural products. It can also help to identify evolutionary histories, inter-organismal interactions, and quantitative information on genomic variability in natural microbial populations. High-quality results currently require state-of-the-art instrumentation such as cell sorters, robotic liquid handlers, DNA sequencers, and a cleanroom. The amount of data to obtain new biological insights requires high-performance information technology with computer clusters for conducting assemblies and analyses of the sequences. Undoubtedly, the future for SC omics is exceptionally bright, but significant technical and conceptual challenges still have to be resolved. In order to expand the range of microorganisms amenable to SC omics, improvements of the methods are necessary. The rapidly growing number of applications that use limited quantities of DNA in genomic detection and analysis highlights the continued need for innovation in the field of DNA amplification and library preparation, e.g., nanoscale library preparation via microfluidics (Kim et al. 2017)**.** This may also result in the omission of the amplification step, or at least its minimization, resulting in less biased sequencing results. In addition, further automation and miniaturization of sequencing processes along with the development of new labeling methods are required for emerging research needs. This will help create a more phylogenetically balanced representation of genomes in databases, which will ultimately help to improve models for computational gene annotation and taxonomic assignment (Woyke et al. 2009; de Jager and Siezen 2011; Wang and Navin 2015). It will also help with the cultivation of microorganisms of interest through facilitating a more informed development of culturing methods by revealing the nutritional needs and metabolic capabilities of the organism (Pratscher et al. 2018)**.**
